# Seasonal, sex variations in vitamin d levels and their association with pulmonary function in children with asthma

**DOI:** 10.3906/sag-1904-112

**Published:** 2019-10-24

**Authors:** Şebnem ÖZDOĞAN

**Affiliations:** 1 Pediatric Pulmonology, Şişli Hamidiye Etfal Research and Training Hospital, İstanbul Turkey

**Keywords:** Vitamin D, asthma, seasonal variation, children

## Abstract

**Background/aim:**

We aimed to examine the seasonal and sex variations in vitamin D levels in children with asthma and their associations with lung function.

**Materials and methods:**

We conducted a cohort study involving children aged 7–17 years old with asthma. Vitamin D levels were obtained and pulmonary function tests (PFTs) were performed in winter months (December, January, and February) and at the end of summer (August, September, and October). Seasonal and sex variations in vitamin D levels and lung function were examined.

**Results:**

A total of 56 children (26 males, mean age: 11.93 ± 1.8) were enrolled. The mean vitamin D level in winter was 13.36 ± 6.31 ng/mL and increased to 22.89 ± 7.83 ng/mL at the end of summer. Vitamin D levels were significantly lower in the female participants (P = 0.002) in winter. There was no difference in vitamin D levels at the end of the summer between the sexes. No correlations were found between vitamin D levels and PFT parameters in winter or at the end of summer.

**Conclusion:**

There are seasonal and sex variations in vitamin D levels in children with asthma. Vitamin D levels do not correlate with lung function.

## 1. Introduction

Vitamin D is an essential substance that plays important roles both in bone metabolism and as an immunomodulatory [1]. Serum 25-OH vitamin D level is the most reliable marker of an individual’s vitamin D level [2]. Factors that affect vitamin D levels include age, sex, exposure to the sun (i.e. latitude, season, time spent outside, and clothing style), skin pigmentation, sunscreen, obesity, and diet [3].

Vitamin D deficiency is pandemic, and more than 1 billion people around the world are thought to have vitamin D deficiency or insufficiency [4]. In developing countries with sunny climates, such as Turkey, the prevalence of vitamin D deficiency has been reported to be 60% in all age groups, and the rates of prevalence are 91% in females and 89% in males during adolescence [5]. The impact of seasonal changes over serum vitamin D has been addressed in several previous studies [6–8]. Lower serum vitamin D level in winter months compared with summer has been shown [6–8].

Although a number of studies have investigated the link between vitamin D and asthma and allergy in recent years, there is still no consensus regarding this issue. Epidemiologic data in most of the reported studies suggest that low serum vitamin D in children with asthma is associated with more symptoms, exacerbations, reduced lung function, increased markers of allergy, and severe disease [9]. Serum 25-OH vitamin D levels were shown to be inversely associated with asthma and there was a direct and significant relationship between vitamin D levels and pulmonary function test (PFT) outcomes in asthmatic children [10]. 

In the present study, we investigated variations in vitamin D levels according to sex and season in asthmatic children and their associations with PFTs. We hypothesized that low vitamin D levels are associated with poor pulmonary function parameters. 

## 2. Materials and methods

### 2.1. Patients

A total of 56 patients (26 males and 30 females) between 7 and 17 years of age who had a persistent asthma diagnosis as per the National asthma education and prevention program (NAEPP) [11] and were followed at pediatric outpatient pulmonology clinics were enrolled in the study. All subjects had been on daily controller with inhaler corticosteroids (ICS). Patients’ vitamin D levels were measured in the winter (December, January, and February) and at the end of summer (August, September, and October), and PFTs were performed at both times. 

Patients with chronic diseases other than asthma (e.g., kidney, liver, endocrine, metabolic, or neurological disorders), those who were taking vitamin D supplements prior to the first phase of our study (i.e. before the winter), and those treated with antiepileptics or steroids that may have influenced vitamin D levels were excluded from the study. Demographic characteristics were recorded. The study was conducted in Istanbul, which is located at a latitude of 40°58′N. 

### 2.2. Vitamin D measurements

In our study, vitamin D levels were measured with liquid chromatography-tandem mass spectrometry (LC-MS/MS) using a Quattro premier XE device as reported to be the gold standards for the measurement of vitamin D [12]. The 25-OH D2 and 25-OH D3 levels were quantified separately and are reported as the total 25-OH vitamin D levels. Vitamin D deficiency in children is defined differently in the endocrinology and pediatrics fields [13,14]. For the purposes of our study, vitamin D levels were considered deficient, inadequate, or normal at ≤20 ng/mL, 20–30 ng/mL, or ≥30 ng/mL, respectively [3,14,15]. Single vitamin D measurements were performed in the winter and at the end of summer. We planned to give the patients with vitamin D levels <30 ng/mL vitamin D supplements; however, only 33 of the 56 patients who had to take vitamin D supplements could be given a single dose of 300,000 IU vitamin D orally. The patients who did not show up regularly for follow-ups and those who did not take the prescribed dose of vitamin D did not receive supplementary therapy. The patients were advised about their diet and sun exposure. Both the patients who received vitamin D supplements and those who did not adhere to the vitamin D therapy were included in the analysis. 

### 2.3. PFT measurements

All PFTs were performed by the same pediatric pulmonary physician. The measurements were performed according to the American Thoracic Society (ATS) standards using an MIR spirometer (MiniSpir®: Medical International Research Srl, Rome, Italy) [16].

The potential confounding factors that may have influenced vitamin D levels were age, sex, BMI, season, recent upper respiratory tract infection, smoking, and passive smoking. 

### 2.4. Statistical analysis

Statistical analyses were performed using the Number Cruncher Statistical System (NCSS) 2007 (Kaysville, Utah, USA) software. Descriptive statistics (mean, standard deviation, median, frequency, ratio, minimum, maximum) were calculated. For the comparison of the quantitative data, Student’s t-test for the intergroup comparison of the parameters with normal distribution and the Mann–Whitney U-test for the intergroup comparison of the parameters outside the normal distribution were performed. The one-way ANOVA test was used in the comparison of three or more groups with normal distribution and the group causing the difference was identified with the Tukey HSD test. The comparison of three or more groups outside the normal distribution was performed using the Kruskal–Wallis test and the group causing the difference was identified with the Mann–Whitney U-test. For comparison of qualitative data, Pearson’s chi-squared test, Fisher–Freeman–Halton exact test, and Yates’s continuity correction test were used. The relationships between the variables were assessed using Pearson’s correlation analysis and Spearman’s correlation analysis. Statistical significance was assessed with values of P < 0.01 and P < 0.05.

## 3. Results

A total of 56 subjects (26 males and 30 females) were investigated in this study. The demographic characteristics, winter and summer vitamin D levels, and pulmonary function test outcomes of the participants are presented in Table 1. The mean age was 11.93 ± 1.8 years. The mean vitamin D level in winter was 13.36 ± 6.3 ng/mL, and the mean vitamin D level at the end of summer was 22.89 ± 7.8 ng/mL. The mean PFT parameters tested in the winter were within normal limits except for the FEV1/FVC ratio. The mean FEV1/FVC ratio in the winter was below 80%. The mean PFT parameters tested in the summer were within normal limits. 

**Table 1:** Demographic characteristics and pulmonary function test parameters of the subjects.

Sex (male/female)	N	%
	26/30	46.4/53.6
	Min–max	± SD
Age (years)	7–17	11.93 ± 1.8
Vitamin D winter (ng/mL)	4.0–28.1	13.36 ± 6.3
Vitamin D summer (ng/mL)	5.2–44.0	22.89 ± 7.8
FEV1 (L) winter	1.36–4.03	2.45 ± 0.53
^#^FEV1 (L) summer	1.67–3.78	2.57 ± 0.51
^&^FEV1% winter	52–132	98.88 ± 15.88
FEV1% summer	53–130	98.55 ± 16.02
^+^FVC (L) winter	2.15–4.28	3,14 ± 0,50
^^^FVC (L) summer	2.19–4.20	3.13 ± 0.53
FVC% winter	77–141	111.20 ± 12.44
FVC% summer	69–141	104.55 ± 12.69
FEV1 / FVC winter	56.20–94.20	77.74 ± 8.96
FEV1 / FVC summer	60.5–99.4	82.24 ± 9.16

The distributions of vitamin D levels measured in the winter and at the end of summer are presented in Figure. While none of the patients had normal vitamin D levels during the winter, 13% (n = 7) of the patients achieved normal levels at the end of summer. Among the patients, 82% (n = 46) exhibited deficient vitamin D levels in the winter and 32% (n = 18) had deficient vitamin D levels at the end of summer. Additionally, 18% (n = 10) had insufficient vitamin D levels in the winter, while 55% (n = 31) had insufficient vitamin D levels at the end of summer. Seasonal and sex variations in vitamin D levels are presented in Table 2. Vitamin D levels were found to be lower in females compared to males during the winter (P = 0.002). No difference was observed between the sexes in the summer (P = 0.20). The mean increases in vitamin D levels were not significantly different between the sexes (P = 0.42). 

**Figure:**
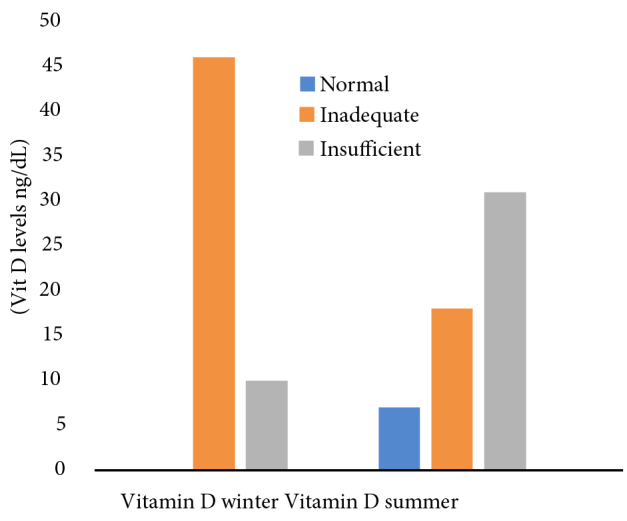
The distributions of vitamin D levels measured in the winter and at the end of summer.

**Table 2:** Seasonal and sex variations in vitamin D levels.

			Vitamin D winter (ng/mL)	Vitamin D summer(ng/mL)	Variations in vitamin D levels (summer–winter)
Sex	Male (n = 26)	Mean ± SD	15.93 ± 5.9	24.35 ± 7.6	8.42 ± 9.1
		min–max (median)	6.8–25.0(15.7)	5.2–42.4 (23.5)	9.5–23.4 (9.1)
	Female(n = 30)	Mean ± SD	11.15 ± 5.8	21.64 ± 7.9	10.50 ± 10.2
		min–max (median)	4–28.1 (9.1)	9.3–44.0 (21.0)	8.9–40.0 (10.6)
		P	^a^0.002**	^b^0.20	^b^0.42

^a^Mann–Whitney U test

^b^Student’s t-test

**P < 0.01

The sex, age, and vitamin D levels in the winter and at the end of summer, and the variations in vitamin D levels among the 33 patients who received vitamin D supplements and 23 who did not receive vitamin D are presented in Table 3. Although the males received greater vitamin D supplementation, no significant difference was observed between the group that received supplements and the group that did not receive supplements in terms of the winter and end-of-summer vitamin D levels (P = 0.93, P = 0.96). The mean variations in vitamin D levels during the winter and summer, and the percentage variation in vitamin D levels did not reveal significant differences between the patients who received supplements and the patients who did not receive supplements (P = 0.99, P = 0.75). 

**Table 3:** Vitamin D supplementation status and clinical features.

		Vitamin D supplementation		P
		Yes	None	
		n (%)	n (%)	
Sex	Male	19 (73.1)	7 (26.9)	^f^0.04
	Female	14 (46.7)	16 (53.3)	
Age (years)	Mean ± SD	11.85 ± 2.0	12.04 ± 1.4	^b^0.72
Vitamin D winter (ng/mL)		13.42 ± 6.1	13.28 ± 6.71	^a^0.93
Vitamin D summer (ng/mL)		22.19 ± 816	22.83 ± 7.51	^b^0.96
Vitamin D variations (summer–winter)		9.51 ± 9.88	9.5 ± 9.66	^b^0.99
Vitamin d variations (%)		116.96 ± 181.99	130.13 ± 163.21	^a^0.75

^a^Mann–Whitney U test_b_Student’s t-test

^f^Yates continuity correction test

^g^Fisher Freeman Halton Test

The associations between vitamin D levels and the PFT parameters are presented in Table 4. No correlations were observed between vitamin D levels measured in the winter and PFT parameters tested in the winter (r = –0.15, –0.20, –0.05, –0.03, and –0.28, P > 0.05). Similarly, no correlations were found between vitamin D levels at the end of summer and PFT tests performed during the same period (r = 0.04, –0.10, 0.06, –0.19, –0.01, P > 0.05). 

**Table 4:** The correlation between vitamin D levels and pulmonary function test parameters.

	Vitamin d winter	
	R	^d^P
FEV 1 winter	–0.15	0.26
FEV 1% winter	–0.20	0.13
FVC winter	–0.05	0.67
FVC% winter	–0.03	0.78
FEV 1/ FVC winter	–0.28	0.33
	Vitamin D summer	
	r	^e^P
FEV 1 summer	0.04	^e^0.76

## 4. Discussion

In the present study, we investigated the seasonal and sex variations in vitamin D levels of children with asthma and their associations with PFTs because vitamin D deficiency is pandemic, and the prevalence of asthma is constantly increasing worldwide. We demonstrated that vitamin D levels are either insufficient or deficient among asthmatic children in winter and also in the majority of children at the end of summer. In winter the levels were even lower in females. Vitamin D levels tended to increase at the end of summer, although there was no difference according to sex. We found no correlations between vitamin D levels and PFT parameters. 

In literature, the risk factors for vitamin D deficiency in children are winter, shorter duration of time spent outside, lower socioeconomic status, adolescence, and lack of vitamin D supplements [7,17]. The increase in vitamin D deficiency with age is attributable to the tendency to spend less time outside and engage in less physical activity. In our study, high rates of vitamin D deficiency were associated with the fact that the majority of subjects were adolescents, spent shorter durations of time outside during winter, and had insufficient dietary vitamin D intake.

Although vitamin D levels exhibited a significant increase at the end of summer, these levels were not sufficient in the majority of patients. We associated the increase in vitamin D levels in this group with vitamin D replacement and the increased protective measures. 

Various studies support the notion that vitamin D levels in Caucasians exhibit seasonal variations. According to these studies, vitamin D levels are highest during the summer and lowest during winter [6–8]. Studies claiming that vitamin D does not exhibit seasonal variations have suggested that vitamin D supplementation suppresses seasonal variation [18]. 

There are reports that have associated low vitamin D levels in asthmatic children with impaired pulmonary function [19] and increased exercise activity [20]. Oral or parenteral steroid treatment is used to treat asthma exacerbations. Long-term or repeated treatment with steroids is known to cause vitamin D deficiency [21]. In a vicious circle, steroids are administered when asthma is poorly managed, which in turn decreases the vitamin D levels and leads to poor management of asthma. Although vitamin D supplementation has been suggested to maintain better management by increasing the antiinflammatory effects of the steroids, further studies are needed to clarify this relationship [21].

In the light of these findings, it remains unclear whether vitamin D plays a direct role in the deterioration of asthma or other allergic diseases and whether it should be a part of the management in these diseases. In a recent study, Babar et al. reported that vitamin D supplementation improves FEV1 significantly at two months in asthmatic adults [22]. In that study it is not clear whether the patients’ vitamin D status and vitamin D supplementation were given at a dose of 50,000 units per day orally. In our study we were unable to demonstrate a correlation between levels of vitamin D and PFTs results in winter or at the end of summer. Although all patients were prescribed vitamin D supplements in winter, some of the subjects did not receive treatment due to poor follow-up and incompliance to the prescribed therapy. When vitamin D levels of both groups at the end of summer were compared, no significant difference was observed. While vitamin D supplementation in asthmatic patients is a controversial subject, another discussion point is whether a regimen of 600 IU daily for 3 months or stoss therapy (300,000 IU once via the oral route) should be used. 

Our study also had certain limitations, including the following: the dietary habits, sun exposure, and use of sunscreen were not investigated, and loss to follow-up occurred as in other cohort studies. 

Although İstanbul has a sunny climate, vitamin D deficiency is rather prevalent. Vitamin D levels of asthmatic patients were low and varied according to season and sex. However, low vitamin D levels were not associated with PFT results. We believe that vitamin D deficiency, an important health problem, is more prevalent than expected in our country, which receives ample sunshine, and that vitamin D supplementation for at-risk groups, including adolescents, should be considered. Female children should be prescribed vitamin D supplements in the winter, and adolescents should be given the opportunity to spend more time outside. 

## Acknowledgments

We wish to thank the children with asthma and their families for taking part in this study.
